# The rice mitochondria proteome and its response during development and to the environment

**DOI:** 10.3389/fpls.2013.00016

**Published:** 2013-02-07

**Authors:** Shaobai Huang, Rachel N. Shingaki-Wells, Nicolas L. Taylor, A. Harvey Millar

**Affiliations:** Australian Research Council Centre of Excellence in Plant Energy Biology and Centre for Comparative Analysis of Biomolecular Networks, The University of Western AustraliaCrawley, WA, Australia

**Keywords:** rice, mitochondrial proteome, mitochondrial proteins, development, stress response

## Abstract

Rice (*Oryza sativa* L.) is both a major crop species and the key model grass for molecular and physiological research. Mitochondria are important in rice, as in all crops, as the main source of ATP for cell maintenance and growth. However, the practical significance of understanding the function of mitochondria in rice is increased by the widespread farming practice of using hybrids to boost rice production. This relies on cytoplasmic male sterile (CMS) lines with abortive pollen caused by dysfunctional mitochondria. We provide an overview of what is known about the mitochondrial proteome of rice seedlings. To date, more than 320 proteins have been identified in purified rice mitochondria using mass spectrometry. The insights from this work include a broad understanding of the major subunits of mitochondrial respiratory complexes and TCA cycle enzymes, carbon and nitrogen metabolism enzymes as well as details of the supporting machinery for biogenesis and the subset of stress-responsive mitochondrial proteins. Many proteins with unknown functions have also been found in rice mitochondria. Proteomic analysis has also revealed the features of rice mitochondrial protein presequences required for mitochondrial targeting, as well as cleavage site features for processing of precursors after import. Changes in the abundance of rice mitochondrial proteins in response to different stresses, especially anoxia and light, are summarized. Future research on quantitative analysis of the rice mitochondrial proteomes at the spatial and developmental level, its response to environmental stresses and recent advances in understanding of the basis of rice CMS systems are highlighted.

## INTRODUCTION

Rice is the one of the key model plants for research and also the major food crop in developing countries. Dramatic increases in rice production have occurred in the past few decades through large scale hybrid rice cultivation using cytoplasmic male sterile (CMS) lines with abortive pollen caused by dysfunctional mitochondria (Eckardt, 2006; Wang et al., 2006). This tremendous advance highlights the significance of understanding rice mitochondrial function. The role and nature of rice mitochondria also takes on special significance due to its early growth in hypoxic or even anoxic environments (Perata and Voesenek, 2007) as well as its need for rapid mitochondrial biogenesis and function during re-oxygenation (Millar et al., 2004; Howell et al., 2007). Mitochondria contain many hundreds of different proteins that initiate or co-ordinate the biochemical processes essential for its function. It is estimated that while only ~300 proteins are components of the respiratory apparatus, up to 2000 proteins are housed in plant mitochondria with the majority encoded in the nucleus and transported into mitochondria as cytosolic precursor proteins by the mitochondrial protein import machinery (Millar et al., 2005; Cui et al., 2011). Because software-based subcellular targeting prediction offers low fidelity in actual subcellular localization (Heazlewood et al., 2005, 2003), direct experimental analysis of mitochondrial proteomes, including that of rice, is required to obtain precise information on which proteins are mitochondrially located. More broad advances in rice proteomics have been well summarized recently (Agrawal and Rakwal, 2011). In this review, recent research on rice mitochondrial purification, contaminant removal, and rice mitochondrial proteomic analysis are discussed. The rice mitochondrial protein composition, functional classifications, and features of mitochondrial protein presequences are summarized. We also discuss the effects of the environment, in particular anoxia and light, on rice mitochondrial proteome composition and how the proteome differs in CMS lines. Finally, we propose future directions for research on the rice mitochondrial proteome.

## PURIFICATION AND PROTEOMIC ANALYSIS OF RICE MITOCHONDRIA

Removal of contaminants, like chloroplasts, from purified rice mitochondria is critical for downstream protein separation and identification of mitochondrial proteins. Classically, differential and gradient centrifugation methods based on size and density have been applied to plant mitochondrial proteomic analysis (Kruft et al., 2001; Millar et al., 2001; Bardel et al., 2002). Using these approaches, mitochondria have been purified on Percoll density gradients from dark-grown rice seedlings (Heazlewood et al., 2003) and from green rice seedlings (Kristensen et al., 2004). The purified mitochondria from dark-grown seedlings were then separated using 2-D IEF/SDS-PAGE, blue native (BN)-PAGE and 122 proteins were identified using LC-MS/MS (Heazlewood et al., 2003). In another similar study that used a sucrose gradient for mitochondrial purification, a set of 112 non-redundant rice mitochondrial proteins were identified after 2-D IEF/SDS-PAGE separation ^1^. (Komatsu, 2005). Comparison of these two studies revealed less than 20% overlap in the two datasets of highly abundant proteins, highlighting the importance of optimized methods for mitochondria purification prior to proteomic analysis.

Free-flow electrophoresis in zone electrophoresis mode (ZE-FFE) can be used to separate organelles based on differential surface charge and this has allowed the comprehensive analysis of *Arabidopsis* organellar proteomes including the exclusion of contaminating proteins through quantitative analysis (Eubel et al., 2007, 2008). The combination of traditional differential and gradient centrifugation with this new FFE separation technique has allowed isolation of highly purified rice mitochondria for proteomic analysis (Huang et al., 2009a). Quantitative analysis using differential in gel electrophoresis (DIGE) and spectral counting have allowed the identification of contaminant proteins removed by FFE purification (Huang et al., 2009a). The purity of isolated mitochondria was >95% based on calculating the number of peptides from contaminant proteins compared to peptides from mitochondrial proteins in these preparations (Huang et al., 2009a). In total, 322 proteins from FFE purified rice mitochondria were identified through the direct analysis of trypsin-digested peptides by LC-MS/MS and gel-based analysis (Huang et al., 2009a). The annotations of rice mitochondrial protein spots on 2-D IEF/SDS/PAGE gel are available online^2^ using the gel-map tool (Klodmann et al., 2011; Senkler and Braun, 2012). Seventy-eight proteins identified previously as components of the rice mitochondrial proteome (Heazlewood et al., 2003) were also in this study. Half of the unconfirmed proteins from Heazlewood et al. (2003) were proteins now predicted to be retrotransposon sequences with unknown function.

## THE PROTEIN COMPOSITION OF RICE MITOCHONDRIA

**FIGURE 1:**
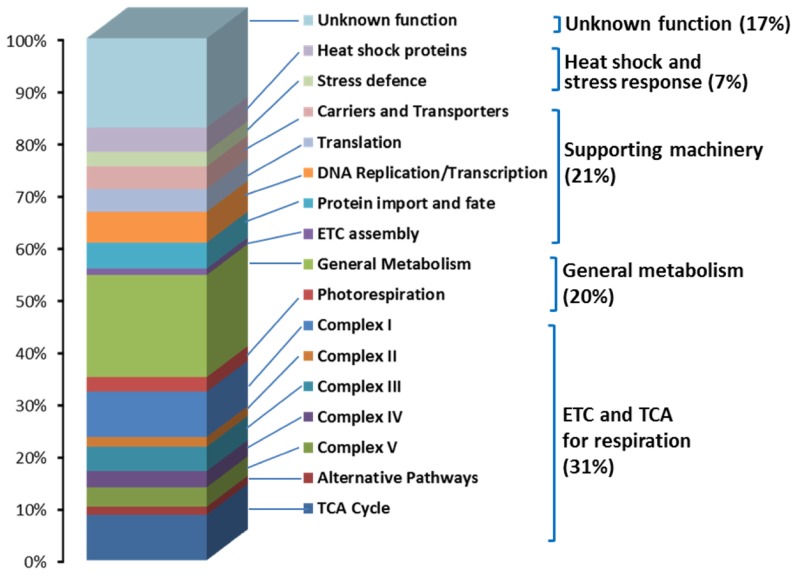
**Functional distribution of 322 identified rice mitochondrial proteins**. Rice mitochondria were purified from 10-day-old dark-grown seedlings using differential and gradient centrifugation combined with surface charged ZE-FEE mode (Huang et al., 2009a). Rice mitochondria proteins were separated using gel-based and non-gel based methods and digested with trypsin before identification using mass spectrometry (Huang et al., 2009a). Rice mitochondria protein data were extracted from Supplemental Table S3 of Huang et al. (2009a)

A refined dataset of 322 proteins allowed us to assess the functional distribution of the rice mitochondrial proteome as shown in **Figure 1**. There are 99 proteins identified as either components of the five oxidative phosphorylation/respiratory complexes or TCA cycle enzymes, representing 31% of the total set (**Figure 1**). The genes encoding electron transport chain (ETC) proteins are highly expressed across all tissues, which is consistent with the fundamental role of mitochondria in energy production throughout the plant. Interestingly, a series of genes encoding TCA cycle components are highly expressed in anthers, suggesting a high energy requirement for metabolism in this tissue (Huang et al., 2009a). There were 64 proteins identified (20% of total set) that are thought to be involved in central carbon and nitrogen metabolism (**Figure 1**), such as the inter-conversion of amino acids, photorespiratory glycine oxidation, synthesis of lipids, vitamins, as well as export of organic acids. Within this group, the identification of a 4-methyl-5-thiazole monophosphate biosynthesis protein (Os01g11880) provided new insight into the involvement of rice mitochondria in the process of thiamine biosynthesis. Furthermore, the highly selective expression of genes for components of photorespiratory glycine oxidation in leaf tissues is consistent with the role of mitochondria in photorespiration during photosynthesis in green tissues (Huang et al., 2009a). Proteins involved in supporting machinery such as those for DNA replication, transcription and translation, protein import and fate, ETC assembly as well as carriers and transporters accounted for 21% of the total number of proteins identified. Thirty-three proteins were listed to be involved in DNA replication, transcription, and translation, and 19 proteins were assigned the protein import and fate category (**Figure 1**). Genes encoding mitochondrial enzymes involved in DNA replication, transcription, and translation, as well as protein import and fate are highly expressed in early germinated rice seeds as well as in suspension culture cells, consistent with their role in mitochondrial biogenesis (Huang et al., 2009a). Fifteen heat shock proteins and 9 putative stress response proteins were also identified (**Figure 1**). A total of 55 proteins (17%) were identified for which no known function has been reported (**Figure 1**).

From the 313 nuclear-encoded rice mitochondrial proteins identified, ~65% were predicted to be located in mitochondria by four different subcellular localization prediction software packages (Huang et al., 2009a). The low fidelity of the prediction software is due in part to the use of a limited number of targeting signals in training sets for these software packages (Heazlewood et al., 2004), which again highlights the importance of building experimental evidence for the mitochondrial location of proteins. The number of identified proteins involved in the ETC and TCA cycle in monocotyledon rice mitochondria is similar to number of identified in *Arabidopsis* mitochondrial datasets (Heazlewood et al., 2004) and the corresponding proteins are also largely conserved (Huang et al., 2011). Proteins involved in supporting machinery and stress response were also conserved between the rice and *Arabidopsis* datasets (Huang et al., 2011). The conservation of the proteomes between these diverse species highlights the fundamental role of mitochondria in energy production and metabolism in plants.

## THE RICE MITOCHONDRIAL PROTEIN PRESEQUENCE AND ITS CLEAVAGE

N-terminal presequences carry the targeting signals required to import nuclear-encoded mitochondrial proteins and these are cleaved off following the import process to generate mature proteins (Zhang and Glaser, 2002). Analysis of the peptides derived from the digestion of mature rice mitochondrial proteins allowed us to experimentally identify cleavage sites and thus determine 52 rice mitochondrial presequences (Huang et al., 2009b). The average length of these presequences is 45 amino acids. The average pI of the first 10 amino acids was 11.8 with a hydrophobicity index of -1.4. Nearly 90% of the presequences were predicted to form α-helices in this region (Huang et al., 2009b). These features are very similar to those observed for *Arabidopsis* mitochondrial proteins (Huang et al., 2009b).

**FIGURE 2:**
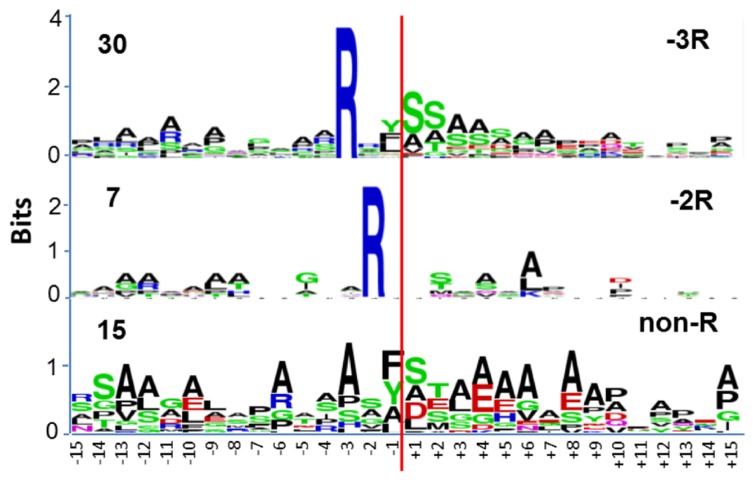
**Sequence logo analysis of 52 rice mitochondria protein precursors at the region of the cleavage site**. The red line indicates the cleavage sites as supported by proteomic evidence. The cleavage sites are grouped into three classes (class II, -3 Arg; Class I -2 Arg; non-conserved Arg, class III). The numbers on the left side represent the total sequences identified in different groups for sequence logo analysis. Data adapted from Huang et al. (2009b).

Amongst the rice mitochondrial presequences three groups of cleavage sites were found: -2 Arg (class I), -3 Arg (class II); and one without any conserved Arg (class III; **Figure 2**). The majority of presequences were -3 Arg (58%) with a smaller contingent of -2 Arg (13%), and a surprisingly large percentage without any conserved arginine (29%; **Figure 2**). In the dominating -3 Arg group, the occurrence of Tyr/Phe/Leu at the -1 position was evenly distributed (**Figure 2**), which differs from the similar *Arabidopsis* -3 Arg group which predominantly features Phe at the -1 position (Huang et al., 2009b). In yeast, an intermediate cleaving peptidase (Icp55, P40051) removes one residue from the presequence after cleavage by the mitochondrial processing peptidase (MPP) when it contains an Arg residue at the -3 position (Vögtle et al., 2009). It is likely that in the mitochondria of rice and *Arabidopsis*, the observed -3 Arg proteins are a consequence of an Icp55-like cleavage, after MPP cutting by an uncharacterized protease (**Figure 2**). Yeast Icp55 (P40051) does have a rice ortholog (Os12g37640; *E* = 2 × e^-91^). We have not found Os12g37640 in the rice mitochondrial protein data set (Huang et al., 2009a), but it is predicted to be located in mitochondria by Target P and Mitoprot II. Future functional analysis of Icp55-like peptidase in plant mitochondria is needed to understand its role in stabilizing mitochondrial proteins following MPP cleavage.

## CHANGES IN THE RICE MITOCHONDRIA PROTEOME DURING ENVIRONMENTAL STRESS AND PLANT DEVELOPMENT

Most rice proteomic analyses in response to environmental stresses have been conducted at the whole tissue level in leaves or leaf sheaths. Mitochondrial proteins contribute only ~2–5% of total cellular protein and this makes them difficult to quantify in whole tissue protein extracts. For example, there no mitochondrial proteins with significant changes in abundance were detected in rice leaves or sheaths under drought (Salekdeh et al., 2002; Ali and Komatsu, 2006) or infected with the fungi *Rhizoctoni solani* (Lee et al., 2006). A few cases, two highly abundant mitochondrial proteins, both glycine dehydrogenase subunits (Os06g40940; Os01g51410), were significantly changed after treatments of heat (Lee et al., 2007), cold (Lee et al., 2009; Yan et al., 2006), and salt (Kim et al., 2005). Isolated single observations of mitochondrial proteins changing in abundance from whole tissue extracts include pyruvate dehydrogenase (Os02g50620), NADH-ubiquinone oxidoreductase 75 kDa protein (Os03g50540), aconitase hydratase (Os08g09200), dihydrolipoyl dehydrogenase (Os01g22520) after treatment with heat (Lee et al., 2007), cold (Lee et al., 2009), and salt (Kim et al., 2005; Chitteti and Peng, 2007). It is clear that to obtain a more detailed picture of the mitochondrial proteome in response to different environmental stresses, purified mitochondria would be required. Using isolated rice mitochondria for protein oxidation analysis, it was found that a number of proteins are oxidized in the matrix *in vivo* and a group of proteins are particularly susceptible to mild oxidation *in vitro* (Kristensen et al., 2004).

The early growth habitat of rice is often hypoxic or even anoxic (Perata and Voesenek, 2007), meaning that the role and nature of rice mitochondria is especially interesting given their central role in respiration. An early study showed that anoxic rice shoots had the ability to synthesize the same range of mitochondrial proteins as aerobic shoots as long as ATP was supplied, which could be provided *in vivo* by glycolytic reactions even in the absence of oxygen (Couée et al., 1992). Analysis of the soluble rice mitochondrial proteome using 2-D IEF/SDS-PAGE gel separation showed no significant difference between samples derived from anoxic and reoxygenated coleoptiles (Millar et al., 2004). However, BN-SDS-PAGE gels of mitochondrial membrane-associated complexes showed a very low abundance of assembled b/c_1_ complex and cytochrome *c* oxidase in anoxic samples and a dramatic increase in the abundance of these complexes after 1 day of air adaptation (Millar et al., 2004). These results suggested that anoxic rice does have the capacity to develop its respiratory machinery but with a discrete and reversible blockage of full mitochondrial biogenesis at Complex III (Millar et al., 2004). Howell et al. (2007) showed that anoxic conditions reduced the efficiency of the general import pathway but not the carrier import pathway in rice mitochondria. Rice mitochondria from anoxic seed embryos 48 h after germination had much lower abundance of TCA cycle enzymes and cytochrome-containing complexes of the respiration chain (Howell et al., 2007). In a whole-cell proteomic analysis, malate dehydrogenase and two ATP synthase subunits were lower in abundance in 6-day-old anoxic coleoptiles compared to similar sized 4-day-old aerated coleoptiles (Shingaki-Wells et al., 2011). The lower abundance of enzymes involved in the TCA cycle or ETC agrees with the previous observation that there is a reduced respiratory capacity in mitochondria isolated from anoxic coleoptiles when compared to aerated or re-oxygenated samples (Millar et al., 2004).

To date the analysis of rice mitochondrial integral membrane proteins has identified seven membrane carrier proteins, one of which was only routinely found in mitochondrial samples from anoxic tissue (Taylor et al., 2010). Further quantitative analysis of the relative abundance of this basic amino acid carrier (BAC; Os10g42299) by QqQ SRM mass spectrometry revealed that Os10g42299 was threefold more abundant in anoxic than in aerated samples (Taylor et al., 2010). Along with the observed anoxic induction of mitochondrial arginase and the accumulation of Arg and Orn, this mitochondrial BAC is likely to play a role in Arg metabolism during O_2_ deprivation (Taylor et al., 2010). Such mitochondrial responses may contribute to the exceptional anoxia tolerance of rice seedlings.

Decreasing the rate of photorespiration has become a key target in the further improvement of rice production (Hibberd et al., 2008). Mitochondria are specifically involved in photorespiration via the oxidation of glycine and the export of serine (Walker and Oliver, 1986). The light responsiveness of mitochondrial functions and the induction of photorespiration that occurs when etiolated rice seedlings are exposed to light was recently investigated using a proteomic and metabolomic approach (Huang et al., 2013). Specific steps in mitochondrial TCA cycle metabolism were decreased under high light which correlates with lower respiration rate (Huang et al., 2013). Light treatment reduced the abundance of mitochondrial enzymes in branched chain amino acid metabolism, correlating with a decrease of the abundance of a range of amino acids after a 24 h light treatment of etiolated shoots (Huang et al., 2013). These results have parallels in the diurnal changes observed in mitochondrial function in *Arabidopsis* shoots (Lee et al., 2010). Significant accumulation of glycine decarboxylase (GDC) P, T subunits and serine hydroxymethyltransferase were observed upon light treatment in rice (Huang et al., 2013), which is similar to what has been observed in pea (Walker and Oliver, 1986; Turner et al., 1993) and *Arabidopsis* (Lee et al., 2010). However, the abundance of the GDC H subunit protein in rice was unchanged by light, and the abundance of GDC L subunit protein was halved under high light. The differential change in the stoichiometry of GDC subunits in rice correlates with a fourfold increase in the photorespiration rate of low light-treated plants compared to those treated with high light (Huang et al., 2013).

Cytoplasmic male sterility is a fundamental part of hybrid rice production and relies on plant lines with pollen-specific defects in mitochondrial function (Eckardt, 2006; Wang et al., 2006). Most CMS-associated genes in rice are chimerics composed of a fragment of a normal mitochondrial gene, encoding small and low abundance mitochondrial membrane proteins, and a novel and disruptive sequence that influences the expression or the function of the gene product (Hanson and Bentolila, 2004; Kubo and Newton, 2008). Quantitative proteomic analysis of CMS-related changes in rice anthers has revealed eight proteins with abundances that are at least twofold lower or higher when comparing YTA (CMS) and YTB (isogenic fertile) lines (Sun et al., 2009). However, none of these were mitochondrially encoded proteins. Further quantitative analysis of the mitochondrial proteomes from 10-day-old rice seedlings has revealed a reduced abundance of specific proteins in mitochondrial complexes, particularly complex V, in the YTA line compared with the YTB line (Liu et al., 2012). Interestingly, a sex determination TASSELSEED-2-like protein (Os07g46920) was found 3.2-fold more abundant in the CMS line (Liu et al., 2012). Analysis of the potential links between the increase in the amount of this protein and jasmonic acid metabolism has identified a lesion in the jasmonic acid synthesis pathway during the development of microspores in CMS plants (Liu et al., 2012).

## FUTURE DIRECTIONS

The plant mitochondrial proteome is a changing entity over time, in different tissues/organs and in response to different environments, as revealed by discoveries made in mitochondrial proteome research of the dicotyledon model plant *Arabidopsis* (Sweetlove et al., 2002; Lee et al., 2011, 2010; Tan et al., 2012). The rice mitochondrial proteome is likely to share these dynamics based on the analysis of rice transcript data for genes encoding mitochondrial proteins (Huang et al., 2009a) as well as the proteome response to anoxia and light as discussed above. Further quantitative analysis of the rice mitochondria proteome will provide an even more detailed picture of the diversification of mitochondrial function at the spatial and developmental levels in this key model monocotyledonous species. A broader understanding of the plasticity of rice mitochondria is particularly important for obtaining more clues on the mechanism of pollen abortion in CMS lines. Furthermore, co-expression analysis will reveal mitochondrial proteins with common functions to provide insights into the regulation of rice mitochondrial biogenesis as well as the respiratory stress response.

## Conflict of Interest Statement

The authors declare that the research was conducted in the absence of any commercial or financial relationships that could be construed as a potential conflict of interest.

## Footnotes

1 http://gene64.dna.affrc.go.jp/RPD

2 www.gelmap.de/oryza
